# Characteristics of persons accused of intimate partner homicide amongst forensic psychiatric observations

**DOI:** 10.4102/sajpsychiatry.v27i0.1675

**Published:** 2021-05-31

**Authors:** Sonali N. Valabdass, Ugasvaree Subramaney, Amanda Edge

**Affiliations:** 1 Department of Psychiatry, School of Clinical Medicine, Faculty of Health Sciences, University of the Witwatersrand, Johannesburg, South Africa

**Keywords:** IPV, IPH, IPH characteristics, IPH perpetrators, IPH perpetrator characteristics, forensic psychiatric observation, IPH risk factors

## Abstract

**Background:**

Intimate partner homicide (IPH) is a global public health problem. One study conducted over 66 countries found that 13.5% of all homicides and 38.6% of female homicides were committed by an intimate partner. In South Africa, there were no published studies that examine alleged perpetrators of IPH that were referred for forensic psychiatric observation.

**Aim:**

To describe the profile of accused persons referred for forensic psychiatric observation for a charge of murder or attempted murder of their intimate partners. Certain characteristics were further examined according to the psychiatric observation outcomes.

**Setting:**

The study was conducted at Sterkfontein Hospital, a forensic psychiatric hospital in Gauteng, South Africa.

**Methods:**

A retrospective record review of accused persons referred for forensic psychiatric observation for a charge of murder or attempted murder of their intimate partners was conducted. The period of the review was 19 years. The definition of intimate partners included current or former spouses and partners, same-sex partners and rejected suitors.

**Results:**

One hundred and sixty-three files, which included forensic psychiatric reports, were reviewed. The findings related to the profile of accused persons and offence characteristics indicated that: (1) history of violent behaviour is prevalent; (2) homicides mostly occur in private homes; (3) knives and firearms are most often used; (4) infidelity, separation and jealousy are common motives; (5) psychotic disorders, personality disorders and substance use disorders feature prominently. A total of 88% of the sample were found fit to stand trial and 82% were found criminally responsible. Factors significantly associated with being found fit to stand trial and criminally responsible following the forensic psychiatric observation were: male gender, having received a tertiary education, employment prior to the offence, earning a salary of more than R10 000, having no previous psychiatric or medical illness, a positive forensic history, previous intimate partner violence (IPV) perpetration, indicating a motive for the homicide, having no psychiatric illness at the time of the offence which would impact fitness to stand trial and criminal responsibility.

Factors significantly associated with being found not fit to stand trial and not criminally responsible following the forensic psychiatric observation were: female gender, having received a primary education, unemployment prior to the offence, having a previous psychiatric or medical illness, no forensic history, no previous IPV perpetration, not indicating a motive for the homicide, having a psychiatric illness at the time of the offence which would impact fitness to stand trial and criminal responsibility.

**Conclusion:**

The characteristics highlighted in this study can contribute to the development of risk assessment tools which can be used to identify likely perpetrators of IPH. Other interventions, for example controlling access to knives and firearms, reducing substance abuse and improving mental health services, are also important in the prevention of IPH.

## Introduction

Intimate partner homicide (IPH), defined as ‘the intentional killing of one’s current or former partner’,^1^ is considered the most extreme form of intimate partner violence (IPV). Such homicides may involve spouses, ex-spouses, current or former partners, or partners of same-sex relationships and include both male and female victims.^1,2^ Fatal IPV can be best understood as ‘an extension of the IPV phenomenon rather than within the scope of general homicide’.^3^

A global study conducted over 66 countries found that 13.5% of all homicides and 38.6% of female homicides were committed by an intimate partner.^4^ A national study of female homicides in South Africa (SA) found that, in 1999 and 2009, approximately 50% of victims were murdered by an intimate partner.^5,6^ This highlights that IPH is a global public health problem that needs to be addressed. In order to curb incidences of IPH, understanding the profiles of these accused persons might assist with identification of potential perpetrators.

Some literature on IPH considers the high prevalence of mental illness amongst perpetrators.^1,2,7,8,9^ In a study that examined 153 alleged perpetrators referred for forensic psychiatric observation for a charge of murder in Illinois, Missouri, Indiana, Colorado or Arizona, 45.8% had a psychiatric diagnosis.^2^ Another study, that was a consecutive case series of all convicted IPH perpetrators in England and Wales between 1997 and 2008, found a 32% lifetime prevalence rate of mental illness.^7^ In a national study conducted in Portugal, 14.3% of alleged IPH perpetrators who were subjected to a forensic psychiatric observation, were found not criminally responsible because of mental illness.^3^ An Italian study showed similar results (12.6%).^10^ In SA, a study that examined alleged homicide perpetrators (of which 44% were alleged IPH perpetrators) referred for forensic psychiatric observation to Weskoppies Psychiatric Hospital, found that 56% had a psychiatric diagnosis at the time of the incident that impacted on criminal responsibility.^11^

Significant gaps exist in the current literature with regard to the profile of accused persons and offence characteristics in cases of IPH referred for forensic psychiatric observation. Despite much research on the general population that commits IPH; few studies have addressed accused persons who are referred for forensic psychiatric observation.

Forensic psychiatric observations are conducted when the court has reason to believe accused persons may be suffering from a mental illness or intellectual disability which may be impacting on the individual’s fitness to stand trial (the ability to comprehend court proceedings) and/or criminal responsibility (the ability to appreciate the wrongfulness of an act and to act in accordance with such an appreciation).^12^

Within the South African context, such accused persons are referred by the court to a psychiatric hospital for a forensic psychiatric observation in terms of sections 77, 78 and 79 of the *Criminal Procedure Act* (CPA) 51 of 1977, as amended.^13,14^ Accused persons undergoing forensic psychiatric observation are referred to as observandi. During the psychiatric observation period, the accused person may undergo several assessments by members of the multi-disciplinary team (psychiatrists, clinical psychologists, occupational therapists, social workers and nurses). These assessments always include psychiatric interviews and physical examinations and may include laboratory tests, psychological assessments, occupational assessments and social worker reports. A final report is completed by the psychiatrist(s) for the court, which comments on the accused person’s diagnosis (if any), their fitness to stand trial and criminal responsibility.

For those who are found not fit to stand trial and/or not criminally responsible, for a serious or major offence (for example murder, attempted murder, sexual assault or assault with intent to cause grievous bodily harm [assault GBH]), the accused is usually admitted to the forensic unit as a state patient in terms of section 42 of the *Mental Health Care Act 17 of 2002* for care, treatment and rehabilitation.^15^

As there is no specific charge of IPH or IPV in SA, this research study focussed on murder and attempted murder of intimate partners in the context of forensic psycho-legal assessments in Johannesburg, SA. Attempted murder was included in this study as the authors wanted to investigate the profile of accused persons who had shown intent to commit murder of their intimate partners regardless of whether the act was successful or not. The authors are of the opinion that the profile of accused persons charged with IPH is more associated with the intent to commit murder rather than with the outcome of the act, and that this warranted the inclusion of attempted murder cases in this study. Persons accused of assault GBH against their intimate partner were not included in this study as the authors could not be confident that these accused persons had demonstrated intent to commit murder and thus the authors believed that their profile would be markedly different to those that were accused of murder or attempted murder of their intimate partners.

## Aims

The primary aim of this study was to describe the socio-demographic, clinical and forensic profile of accused persons referred for forensic psychiatric observation, under the CPA, to Sterkfontein Hospital for a charge of murder or attempted murder of an intimate partner. The study also sought to examine offence characteristics and to describe the outcomes of the forensic psychiatric observation. The accused and offence characteristics, as well as diagnosis, were further examined according to the categories of psychiatric observation outcomes, that is, whether accused persons were found to be either fit to stand trial or not fit to stand trial and criminally responsible or not criminally responsible.

## Methods

### Study design and setting

This was a retrospective record review of accused persons referred for forensic psychiatric observation to Sterkfontein Hospital with a charge of murder or attempted murder during a 19-year period, from 01 January 2000 to 31 December 2018.

The study was conducted at the forensic section of Sterkfontein Hospital, a tertiary psychiatric hospital providing both general and forensic psychiatric services, situated in Gauteng, SA.

### Study population

The sample included records of all adult (age ≥ 18 years) males and females referred as observandi from 01 January 2000 to 31 December 2018 for the charges of murder or attempted murder of their intimate partners. The definition of intimate partners included current or former spouses and partners, same-sex partners and rejected suitors. The definition of IPH includes those homicides that were successful and those that were unsuccessful, that is, files included in this study were of those accused persons referred for forensic psychiatric observation with a charge of murder or attempted murder of their intimate partner.

### Data collection

Case files for IPH were identified via the forensic unit admission register, psychiatric reports and clinical records. Data collection took place between January 2019 and June 2019. Data were collected from psychiatric reports and clinical records. Data were captured using a data collection sheet which included socio-demographic, clinical, forensic, offence and psychiatric observation factors.

### Data analysis

All statistical analyses were conducted using Python (Scipy.stats module; https://docs.scipy.org/doc/scipy/reference/stats). All tests were two-tailed probability values. Categorical variables were summarised using frequency tables. Fitness to stand trial and criminal responsibility classifications were compared against accused characteristics, offence characteristics and diagnosis. Pearson’s chi-squared test was used to determine statistical significance. A *p*-value of < 0.05 was considered significant.

### Ethical considerations

The protocol was approved by the University of the Witwatersrand’s Human Research Ethics Committee (clearance certificate number: M180530). Permission to use Sterkfontein Hospital as the site for the research was granted by the hospital’s research committee. All data were collected by the primary investigator who was responsible for ensuring the anonymity, confidentiality and security of data obtained.

## Results

### Profile of accused persons

A total of 145 male and 18 female accused persons were included in this study (Table 1). Of these, 44% were unemployed. Thirty-three accused persons had a previous psychiatric illness (18 described a mood disorder, 14 a psychotic disorder and 1 a personality disorder [PD]). Fifty-seven accused persons were found to have one or more medical illnesses and 108 accused persons admitted to using one or more substances. Ethanol and cannabis were the most commonly reported substances used. Ten per cent of accused persons reported that they had experienced childhood trauma. Thirty-seven per cent reported a forensic history, with a few accused persons reporting more than one charge. Of the sample, 26% were previous perpetrators of IPV. Previous IPV perpetration was confirmed by the accused person and/or collateral reports.

**TABLE 1:** Socio-demographic, clinical and forensic profile of accused persons.

Characteristics	*N*	%
**Age**		
18 to 30	50	31
31 to 40	52	32
Over 40	61	37
**Gender**		
Female	18	11
Male	145	89
**Marital status (at the time of the offence)**		
Single	73	45
Married	59	36
Separated	19	12
Divorced	12	7
**Cohabiting**		
No	79	48
Yes	84	52
**Schooling**		
Mainstream	156	96
Special	3	2
No formal education	4	2
**Highest level of education**		
No formal education	4	2
Primary education	20	12
Secondary education	91	56
Tertiary education	48	29
**Employment status**		
Employed	91	56
Unemployed not on a disability grant	65	40
Unemployed on a disability grant	7	4
**Salary**		
R0 – R5000	4	2
R5000 – R10 000	28	17
> R10 000	59	36
**Previous psychiatric illness**		
No	130	80
Yes	33	20
**Previous psychiatric diagnosis (DSM-IV-TR)**		
Antisocial personality disorder	1	1
Schizophrenia	10	6
Schizoaffective disorder	1	1
Psychotic disorder due to a general medical condition	2	1
Substance-induced psychotic disorder	1	1
Major depressive disorder	13	8
Mood disorder due to a general medical condition	2	1
Bipolar I disorder	2	1
Bipolar II disorder	1	1
**Medical illness**		
No	106	65
Yes	57	35
**Medical diagnosis**		
Previous head trauma	29	18
Epilepsy	21	13
Diabetes mellitus	10	6
HIV	6	4
**History of substance use**		
No	55	34
Yes	108	66
**Substance**		
Ethanol	79	48
Cannabis	46	28
Nicotine	27	17
Stimulants	11	7
**Childhood trauma**		
No	146	90
Yes	17	10
**Nature of childhood trauma**		
Physical and/or sexual abuse	10	6
Witnessed domestic violence	5	3
Both of the above	1	1
Other	1	1
**Forensic history**		
No	103	63
Yes	60	37
**Previous charge**		
Murder	4	2
Attempted murder	5	3
Assault with intent to cause grievous bodily harm	26	16
Domestic violence	2	1
Protection order contravention	10	6
Robbery	20	12
Malicious damage to property	1	1
**History of violent behaviour**		
No	86	53
Yes	77	47
**Perpetrator of domestic violence**		
Indicated – no	34	21
Indicated – yes	42	26
Not indicated	87	53
**Victim of domestic violence**		
No	156	96
Yes	7	4

DSM-IV-TR, Diagnostic and Statistical Manual of Mental Disorders, 4th Edition, Text Revision.

### Offence characteristics

The vast majority of the victims were the current spouse or partner of the accused (Table 2). Stabbing (using a knife) was the most common killing method, followed by gunshot. The majority of offences occurred at a residence.

**TABLE 2:** Offence characteristics.

Characteristics	*N*	%
**Nature of the charge**		
Murder	124	76
Attempted murder	39	24
**Relationship to the accused**		
Spouse	75	46
Partner	65	40
Ex-partner	10	6
Ex-spouse	5	3
Fiancé	4	2
Rejected suitor	4	2
**Killing method**		
Stabbing	76	47
Gunshot	43	26
Strangulation	16	10
Blunt trauma	13	8
Arson	4	2
Poisoning	1	1
Other	6	4
Mixed	4	2
**Murder weapon**		
Knife	70	43
Firearm	43	26
Bodily force	14	9
Fire	4	2
Poison	1	1
Other	27	17
Mixed	4	2
**Setting**		
Residence	128	79
Street	14	9
Other	21	13
**Substance use at the time of offence**		
No	112	69
Yes	51	31
**Motive**		
Indicated	115	71
Not indicated	48	29
**Nature of the motive**		
Rage	51	31
Infidelity	36	22
Separation	35	21
Jealousy	21	13
Self-defence	11	7
Possessiveness	3	2
Financial benefit	1	1
Retaliation	1	1

Sixty-nine per cent of accused persons reported to not have used a substance at the time of the offence. Considerably more accused persons indicated a motive. Of those that indicated a motive, rage, infidelity and separation were the most common.

### Psychiatric observation outcomes

The Diagnostic and Statistical Manual of Mental Disorders, 4th Edition, Text Revision (DSM-IV-TR) classification system was used in this study. Half of the sample were found to have a psychiatric diagnosis (Table 3). Of these, some were found to have more than one diagnosis. Overall, 18% had a substance use disorder (SUD), 15% had a PD, 15% had a psychotic disorder, 9% had a mood disorder, 3% had dementia and 1% had mental retardation. Considerably more observandi were found fit to stand trial than not fit to stand trial and considerably more accused persons were found criminally responsible than not criminally responsible.

**TABLE 3:** Psychiatric observation outcomes.

Characteristics	*N*	%
**Psychiatric diagnosis**		
Not present	82	50
Present	81	50
**Psychiatric diagnosis (DSM-IV-TR)**		
Antisocial personality disorder	20	12
Borderline personality disorder	3	2
Narcissistic personality disorder	1	1
Dependent personality disorder	1	1
Substance use disorder	29	18
Schizophrenia	11	7
Schizoaffective disorder	1	1
Psychotic disorder not otherwise specified	2	1
Psychotic disorder due to a general medical condition	9	6
Substance-induced psychotic disorder	1	1
Major depressive disorder	12	7
Mood disorder due to a general medical condition	2	1
Mental retardation	2	1
Dementia	5	3
**Fitness to stand trial**		
Fit	143	88
Not fit	20	12
**Criminal responsibility**		
Criminally responsible	134	82
Not criminally responsible	29	18

DSM-IV-TR, Diagnostic and Statistical Manual of Mental Disorders, 4th Edition, Text Revision.

### Fitness to stand trial and criminal responsibility

Gender was found to be a significant predictor for fitness to stand trial and criminal responsibility (Table 4).

**TABLE 4:** Accused and offence characteristics and diagnosis according to the categories of psychiatric observation outcomes with *p* values.

Characteristics	*N*	Fitness to stand trial		Criminal responsibility	
		Not fit (%)	*p*	Not responsible (%)	*p*
**Age**			0.14		0.09
18 to 30	50	12		14	
31 to 40	52	6		12	
Over 40	61	18		26	
**Gender**			< 0.01*		< 0.01*
Female	18	44		56	
Male	145	8		13	
**Marital status (at the time of the offence)**			0.75		0.53
Single	73	14		15	
Married	59	12		22	
Separated	19	5		11	
Divorced	12	17		25	
**Highest level of education**			0.02*		0.01*
No formal education	4	25		50	
Primary education	20	30		35	
Secondary education	91	12		19	
Tertiary education	48	4		6	
**Employment status**			< 0.01*		0.02*
Employed	91	4		11	
Unemployed not on a disability grant	65	20		25	
Unemployed on a disability grant	7	43		43	
**Salary**			< 0.01*		0.03*
R0 – R5000	4	0		25	
R5000 – R10 000	28	14		18	
> R10 000	59	0		7	
**Previous psychiatric illness**			< 0.01*		0.02*
No	130	8		14	
Yes	33	30		33	
**Previous psychiatric diagnosis**			< 0.01*		< 0.01*
Personality disorder	1	0		0	
Psychotic disorder	14	64		71	
Mood disorder	18	6		6	
**Medical illness**			0.01*		< 0.01*
No	106	7		10	
Yes	57	23		32	
**Medical diagnosis**					
Previous head trauma	29	24	0.07	31	0.07
Epilepsy	21	24	0.17	38	0.02*
HIV	6	33	0.33	50	0.12
**History of substance use**			0.38		0.11
No	55	16		25	
Yes	108	10		14	
**Childhood trauma**			0.75		0.73
No	146	12		18	
Yes	17	12		12	
**Nature of childhood trauma**			0.82		0.81
Physical and/or sexual abuse	10	20		20	
Witnessed domestic violence	5	0		0	
Both of the above	1	0		0	
Other	1	0		0	
**Forensic history**			0.06		0.03*
No	103	17		23	
Yes	60	5		8	
**History of violent behaviour**			0.06		0.19
No	86	17		22	
Yes	77	6		13	
**Perpetrator of domestic violence**			< 0.01*		< 0.01*
Indicated – no	34	32		47	
Indicated – yes	42	0		5	
Not indicated	87	10		13	
**Victim of domestic violence**			0.05		0.21
No	156	11		17	
Yes	7	43		43	
**Substance use at the time of offence**			0.70		0.80
No	112	13		19	
Yes	51	10		16	
**Motive**			0.02*		< 0.01*
Indicated	115	8		11	
Not indicated	48	23		33	
**Psychiatric diagnosis**			< 0.01*		< 0.01*
Not present	82	0		1	
Present	81	25		35	
**Psychiatric diagnosis**					
Personality disorder	25	0	0.09	4	0.09
Substance use disorder	29	10	0.97	17	0.86
Psychotic disorder	24	67	< 0.01*	83	< 0.01*
Mood disorder	14	7	0.85	21	0.99
Mental retardation	2	0	0.58	0	0.79
Dementia	5	60	0.01*	80	< 0.01*

* , statistically significant.

Males were more likely to be found fit and criminally responsible whereas females were more likely to be found not fit and not criminally responsible.

The level of education completed also had a significant association with fitness to stand trial and criminal responsibility. Accused persons who achieved lower levels of education were more likely to be found not fit and not criminally responsible. Those who had obtained a tertiary education were more likely to be found fit and criminally responsible.

Employment status and salary were significantly associated with fitness to stand trial and criminal responsibility. Accused persons who were employed were more likely to be found fit and criminally responsible. Accused persons who earned more than R10 000 were more likely to be found fit and criminally responsible.

Previous psychiatric illness was found to be a significant predictor for fitness to stand trial and criminal responsibility. Accused persons with a previous psychiatric illness, particularly a psychotic disorder, were more likely to be found not fit and not criminally responsible compared to those without a previous psychiatric diagnosis who were more likely to be found fit and criminally responsible. Those diagnosed with a previous mood disorder were more likely to be found fit and criminally responsible.

Those with a medical illness were more likely to be found not fit and not criminally responsible compared to those without a medical illness who were more likely to be found fit and criminally responsible. Accused persons with a diagnosis of epilepsy were more likely to be found not criminally responsible.

Forensic history was significantly associated with criminal responsibility but not fitness to stand trial. Accused persons with a forensic history were more likely to be found criminally responsible compared to those with no forensic history, who were more likely to be found not criminally responsible.

A history of IPV perpetration and reporting a motive were significantly associated with fitness to stand trial and criminal responsibility. Accused persons with a history of committing previous IPV were more likely to be found fit and criminally responsible compared to those accused persons who were not previous IPV perpetrators, who were more likely to be found not fit and not criminally responsible. Accused persons who indicated a motive were more likely to be found fit and criminally responsible.

The presence of a psychiatric diagnosis at the time of the offence was significantly associated with fitness to stand trial and criminal responsibility. Having psychopathology in keeping with a psychiatric diagnosis, particularly a psychotic disorder or dementia, at the time of the offence, made it more likely for accused persons to be found not fit and not criminally responsible.

## Discussion

This study concurred with the findings in the literature that perpetrators of IPH are predominantly men.^2,7,8,9,16,17,18^

Some studies indicate that approximately half of male perpetrators of IPH had not completed high school and the majority of female offenders have limited educational achievements.^19,20^

This is contrary to our findings which showed that 14% of the sample had not completed secondary education.

A high percentage of accused persons (44%) were unemployed at the time of the offence, a finding consistent with those of most studies.^5,7,19,21,22,23^ The finding that there is an association between employment status and fitness to stand trial corresponds with the findings of another South African study.^24^

In a study that examined characteristics of IPH perpetrators, mental illness was rarely diagnosed before the incident,^17^ contrary to a Dutch study which showed that 59% of offenders had previous contact with psychiatric services.^23^ Our study showed that 20% of the sample had been diagnosed with a mental illness prior to the offence.

Few studies have addressed the presence of medical illnesses amongst IPH perpetrators. Bourget and Gagné reported that 64% of women and 43% of men in their sample had chronic illnesses.^8^ In Hanlon and colleagues’ study, 11% of the sample had epilepsy,^2^ which is similar to our results of 13% having epilepsy. The importance of epilepsy in this context warrants further study, particularly preictal, ictal and postictal psychopathology.

Literature suggests childhood trauma plays a significant role in the risk of IPH perpetration.^23,25,26^ Childhood trauma can include physical and/or sexual abuse or witnessing violence between parents. Putkonen and colleagues found that 61% of female homicide offenders and 39% of male homicide offenders had experienced physical violence in their family.^21^ However, only 10% of our sample reported childhood trauma. Given the important role that adverse childhood events play in the risk of IPH perpetration, the low incidence is likely because of either under-reporting by the observandi or a recording omission by the original assessors, who may have neglected to enquire about childhood trauma at the time.

In this study, 37% of alleged perpetrators had a forensic history and 47% had a history of violent behaviour. These findings were similar to those in the literature which shows that approximately 25% – 50% of all male IPH perpetrators have been imprisoned for a previous brutal crime.^1,2,3,9,23^ Female IPH offenders show less previous criminality compared to their male counterparts.^8,19,21^

In SA, domestic violence is quoted as the main causal factor that results in IPHs.^20^ It is very uncommon for a fatal act of violence against a partner to be the first occurrence of IPV.^3,5,10,23,25^ In Leth’s study, 50% of victims had experienced previous IPV.^18^ Whilst spousal homicides often occur in the setting of IPV, it is important to consider this in the context of gender. Literature consistently indicates that:

[*M*]ale perpetrators are likely to have subjected their partner to previous IPV, and are more likely to murder them following an escalation of violence, whereas women are more likely to kill in self-defence, as an extreme reaction to their victimisation, and/or to protect children.^9,18^

This is further supported by the findings from two national studies conducted from 2003 to 2015 in North America.^17,27^ In our study, however, only 26% of the sample reported being previous perpetrators of IPV. It is possible that this percentage is significantly under-reported as the records mainly consisted of information obtained from the alleged perpetrators’ account and accused persons may have withheld or denied certain information in order to protect their reputation and avoid incrimination. Additionally, no victim characteristics were examined in this study, such as forensic autopsies or police reports, which may have provided more objective information.

Our study found a similar proportion of victims that were killed by their spouses (46%), in comparison to those that were killed by a non-marital partner (42%). This is contradictory to the literature which shows that IPH occurs more frequently within common-law relationships than those in marital relationships.^6,8,25^

Alleged perpetrators killed their intimate partners most often in private residences, using a knife or firearm, which is supported by previous research.^2,3,6,8,9,17,18,19,20,25,27^

Despite the high incidences of substance use in cases of non-fatal IPV, and a study indicating that a significant relationship exists between male perpetrators’ alcohol abuse and violence against intimate female partners,^28^ some studies reveal that most IPH perpetrators did not use alcohol or drugs at the time of the homicide, in spite of their normally high substance abuse rates.^1,2,8^ This was similar to our findings which showed that 69% of the sample reported not using substances at the time of the offence. This could be because of under-reporting in a forensic environment.

Women rarely kill an intimate partner after the couple has separated but men are at greater risk of perpetrating IPH when separation of the relationship has occurred or is imminent.^1,3,9,18,23,25,26^ A Portuguese study found that most women were murdered by ex-partners within a year of separation, highlighting that a significant risk persists even after the couple’s separation.^3^ Jealousy and infidelity are common motives for men to commit IPH.^3,17,18,22,25^ Our study was consistent with the literature which showed rage, infidelity and separation as the most common motives for murder.

Our study showed that 50% of the sample were found to have a psychiatric diagnosis at the time of the offence.

These results, however, must be interpreted with caution as PDs and SUDs were included in this category and these disorders alone do not impact fitness to stand trial and criminal responsibility. It is important to note, however, that PDs and SUDs may have an impact on IPV perpetration at large. Following forensic psychiatric observation (for IPH), studies indicate that psychotic disorders are most common, followed by mood disorders and anxiety disorders.^2,11,23^

Our study found similar results: 15% had a psychotic disorder and 9% a mood disorder. Oram and colleagues, however, found contradictory results in that affective disorders were most common.^7^ Our findings resembled the literature in that there is a high prevalence of PDs in this population, particularly cluster B pathology (borderline, narcissistic, histrionic and antisocial).^2,10,23,25,26^ Research indicates that approximately 10% – 20% of IPH perpetrators have a lifetime primary diagnosis of substance dependence.^3,23^ Our study showed similar findings with 18% of accused persons being found to have an SUD.

Multiple studies revealed that 14% – 20% of alleged IPH perpetrators, referred for forensic psychiatric observation, were deemed not criminally responsible because of the presence of a psychiatric disorder.^3,7,19,21^ Our study showed similar results (18%). This reinforces the need to refer accused persons of IPH for forensic psychiatric observation.

## Limitations

The study’s retrospective nature is a limitation, in that data may not always be complete and information gathered from others’ notes also has the potential to be inaccurate. Another limitation is that accused persons with PDs or SUDs were included amongst those individuals who were found to have ‘severe’ psychiatric illness whose fitness to stand trial and criminal responsibility were impacted. Additionally, these are alleged IPH perpetrators (who are still awaiting trial) so one has to be wary of drawing conclusions about actual convicted perpetrators. Assault GBH of an intimate partner was not included in the analyses, and the authors acknowledge the severe consequences of pervasive assault GBH over time which may be a precursor to attempted murder and murder. This warrants further study.

## Conclusion

This is the first South African published study examining IPH within the context of forensic psychiatric observations. The primary aim of this study was to describe the socio-demographic, clinical and forensic profile of accused persons referred for forensic psychiatric observation, under the CPA, for a charge of IPH. The study also sought to examine offence characteristics and to describe the outcomes of the forensic psychiatric observation. The accused and offence characteristics, as well as diagnosis, were further examined according to the categories of psychiatric observation outcomes, that is, fitness to stand trial and criminal responsibility.

The main findings of this study were: (1) history of violent behaviour is prevalent; (2) homicides mostly occur in private homes; (3) knives and firearms are most often used; (4) infidelity, separation and jealousy are common motives; (5) psychotic disorders, PDs and SUDs feature prominently. Childhood trauma and previous IPV perpetration did not feature prominently. It is essential that an understanding of childhood trauma and IPV perpetration in the context of IPH is pursued, especially in developing countries, so that data can be compared globally. Furthermore, given the small number of alleged female perpetrators, we were not able to explore differences by gender. Future research is needed to examine gender differences amongst IPH perpetrators in SA.

From this study, the ‘typical’ fit and criminally responsible alleged perpetrator of IPH is a male who would have attained a tertiary education, would be employed prior to the offence and earning more than R10 000 a month. He would have no previous mental illness or medical illness but would have a positive forensic history. He would have a history of IPV perpetration and would indicate a motive for the homicide. Following the forensic psychiatric observation, he would not be found to have a psychiatric illness that impacted fitness to stand trial and criminal responsibility but may have a diagnosis of PD and/or SUD.

The ‘typical’ not fit and not criminally responsible alleged perpetrator of IPH is a female who would have attained only a primary education. She would be unemployed prior to the offence and have a history of previous mental illness. She may have a medical illness but would have no forensic history. She would not have a history of IPV perpetration and would not indicate a motive for the homicide. Following the forensic psychiatric observation, she would be found to have a psychiatric illness that impacted fitness to stand trial and criminal responsibility such as a psychotic disorder or dementia.

The study highlights multiple risk factors in those who are accused of IPH and are sent for forensic psychiatric observation. This information is valuable in that it can assist in preventing IPH, through the development of risk assessment tools which can be used for identification of likely perpetrators. Other interventions such as monitoring access to weapons such as knives and firearms, interventions to reduce alcohol and substance abuse and improving mental health services should all be addressed in order to reduce the incidence of IPH.

## References

[1] Kivisto AJ. Male perpetrators of intimate partner homicide: A review and proposed typology. J Am Acad Psychiatry Law. 2015;43(3):300–312. 26438808

[2] Hanlon RE, Brook M, Demery JA, Cunningham MD. Domestic homicide: Neuropsychological profiles of murderers who kill family members and intimate partners. J Forensic Sci. 2016;61(1):163–170. 10.1111/1556-4029.12908 26292990

[3] Pereira AR, Vieira DN, Magalhães T. Fatal intimate partner violence against women in Portugal: A forensic medical national study. J Forensic Leg Med. 2013;20(8):1099–1107. 10.1016/j.jflm.2013.09.015 24237830

[4] Stöckl H, Devries K, Rotstein A, et al. The global prevalence of intimate partner homicide: A systematic review. Lancet. 2013;382(9895):859–865. 10.1016/S0140-6736(13)61030-2 23791474

[5] Abrahams N, Mathews S, Martin LJ, Lombard C, Jewkes R. Intimate partner femicide in South Africa in 1999 and 2009. PLoS Med. 2013;10(4):e1001412. 10.1371/journal.pmed.1001412 23565064 PMC3614499

[6] Abrahams N, Jewkes R, Martin LJ, Mathews S, Vetten L, Lombard C. Mortality of women from intimate partner violence in South Africa: A national epidemiological study. Violence Vict. 2009;24(4):546–556. 10.1891/0886-6708.24.4.546 19694357

[7] Oram S, Flynn SM, Shaw J, Appleby L, Howard LM. Mental illness and domestic homicide: A population-based descriptive study. Psychiatr Serv. 2013;64(10):1006–1011. 10.1176/appi.ps.201200484 23820784

[8] Bourget D, Gagné P. Women who kill their mates. Behav Sci Law. 2012;30(5):598–614. 10.1002/bsl.2033 23015414

[9] Sabri B, Campbell JC, Dabby FC. Gender differences in intimate partner homicides among ethnic subgroups of Asians. Violence Against Women. 2016;22(4):432–453. 10.1177/1077801215604743 26391620 PMC4602399

[10] Carabellese F, Tamma M, La Tegola D, Candelli C, Catanesi R. Women victims of violent partners: The Italian situation amid culture and psychopathology. J Forensic Sci. 2014;59(2):533–539. 10.1111/1556-4029.12347 24745084

[11] Sussman P, Kotze C. Psychiatric features in perpetrators of homicide-unsuccessful suicide at Weskoppies Hospital in a 5-year period. S Afr J Psychiatr. 2013;19(1):15–18. 10.4102/sajpsychiatry.v19i1.384

[12] Kaliski S. Psycholegal assessment in South Africa. Cape Town: Oxford University Press; 2006.

[13] South Africa. Criminal Procedure Act No. 51 of 1977. Government Gazette, 6 May 1977, Vol. 143, No. 5532.

[14] South Africa. Criminal Procedure Amendment Act No. 4 of 2017. Government Gazette, 29 June 2017, Vol. 624, No. 40946.

[15] South Africa. Mental Health Care Act No. 17 of 2002. Government Gazette, 6 November 2002, Vol. 449, No. 24024.

[16] Eriksson L, Mazerolle P. A general strain theory of intimate partner homicide. Aggress Violent Behav. 2013;18(5):462–470. 10.1016/j.avb.2013.07.002

[17] Velopulos CG, Carmichael H, Zakrison TL, Crandall M. Comparison of male and female victims of intimate partner homicide and bidirectionality – An analysis of the national violent death reporting system. J Trauma Acute Care Surg. 2019;87(2):331–336. 10.1097/TA.0000000000002276 31348402

[18] Leth PM. Intimate partner homicide. Forensic Sci Med Pathol. 2009;5(3):199–203. 10.1007/s12024-009-9097-5 19598012

[19] Hellen F, Lange-Asschenfeldt C, Ritz-Timme S, Verhülsdonk S, Hartung B. How could she? Psychosocial analysis of ten homicide cases committed by women. J Forensic Leg Med. 2015;36(1):25–31. 10.1016/j.jflm.2015.08.007 26355562

[20] Hesselink A, Dastile P. A criminological assessment on South African women who murdered their intimate male partners. J Psychol Afr. 2015;25(4):335–344. 10.1002/cbm.782

[21] Putkonen H, Weizmann-Henelius G, Lindberg N, Rovamo T, Häkkänen-nyholm H. Gender differences in homicide offenders’ criminal career, substance abuse and mental health care. A nationwide register-based study of Finnish homicide offenders 1995–2004. Crim Behav Ment Health. 2011;21(1):51–62. 10.1002/cbm.782 20603817

[22] Mathews S, Abrahams N, Jewkes R, Martin LJ, Lombard C, Vetten L. Intimate femicide-suicide in South Africa: a cross-sectional study. Bull World Health Organ. 2008;86(7):552–558. 10.2471/BLT.07.043786 18670666 PMC2647481

[23] Liem M, Koenraadt F. Familicide: A comparison with spousal and child homicide by mentally disordered perpetrators. Crim Behav Ment Health. 2008;18(5):306–318. 10.1002/cbm.710 19072934

[24] Houidi A, Paruk S, Sartorius B. Forensic psychiatric assessment process and outcome in state patients in KwaZulu-Natal, South Africa. S Afr J Psychiatry. 2018;24(0):a1142. 10.4102/sajpsychiatry.v24i0.1142 PMC6138210 30263219

[25] Aldridge M, Browne K. Perpetrators of spousal homicide: A review. Trauma Violence Abuse. 2003;4(3):265–276. 10.1177/1524838003004003005 14697126

[26] Elisha E, Idisis Y, Timor U, Addad M. Typology of intimate partner homicide: Personal, interpersonal, and environmental characteristics of men who murdered their female intimate partner. Int J Offender Ther Comp Criminol. 2010;54(4):494–516. 10.1177/0306624X09338379 19531601

[27] Rizo CF, Mennicke A, Deinse TV. Characteristics and factors associated with intimate partner violence-related homicide post-release from jail or prison. J Interpers Violence. 2019;1–28. 10.1177/0886260519888195 31718399

[28] Sharps PW, Campbell J, Campbell D, Gary F, Webster D. The role of alcohol use in intimate partner femicide. Am J Addict. 2001;10(2):122–135. 10.1080/105504901750227787 11444155

